# QTL underlying some agronomic traits in barley detected by SNP markers

**DOI:** 10.1186/s12863-016-0409-y

**Published:** 2016-07-07

**Authors:** Jibin Wang, Genlou Sun, Xifeng Ren, Chengdao Li, Lipan Liu, Qifei Wang, Binbin Du, Dongfa Sun

**Affiliations:** College of Plant Science and Technology, Huazhong Agricultural University, Wuhan, 430070 China; Biology Department, Saint Mary’s University, 923 Robie Street, Halifax, NS B3H 3C3 Canada; Department of Agriculture & Food/Agricultural Research Western Australia, 3 Baron-Hay Court, South Perth, WA 6155 Australia; Hubei Collaborative Innovation Center for Grain Industry, Jingzhou, 434025 Hubei China

**Keywords:** Barley, High density linkage map, Single nucleotide polymorphism, Grain yield, Agronomic traits

## Abstract

**Background:**

Increasing the yield of barley (*Hordeum vulgare* L.) is a main breeding goal in developing barley cultivars. A high density genetic linkage map containing 1894 SNP and 68 SSR markers covering 1375.8 cM was constructed and used for mapping quantitative traits. A late-generation double haploid population (DH) derived from the Huaai 11 × Huadamai 6 cross was used to identify QTLs and QTL × environment interactions for ten traits affecting grain yield including length of main spike (MSL), spikelet number on main spike (SMS), spikelet number per plant (SLP), grain number per plant (GP), grain weight per plant (GWP), grain number per spike (GS), thousand grain weight (TGW), grain weight per spike (GWS), spike density (SPD) and spike number per plant (SP).

**Results:**

In single environment analysis using composite interval mapping (CIM), a total of 221 QTLs underlying the ten traits were detected in five consecutive years (2009–2013). The QTLs detected in each year were 50, 48, 41, 41 and 41 for the year 2009 to 2013. The QTLs associated with these traits were generally clustered on chromosome 2H, 4H and 7H.

In multi-environment analysis, a total of 111 significant QTLs including 18 for MSL, 16 for SMS, 15 for SPD, 5 for SP, 4 for SLP, 14 for TGW, 5 for GP, 11 for GS, 8 for GWP, and 15 for GWS were detected in the five years. Most QTLs showed significant QTL × environment interactions (QEI), nine QTLs (*qIMSL3-1, qIMSL4-1, qIMSL4-2, qIMSL6-1, qISMS7-1, qISPD2-7, qISPD7-1, qITGW3-1* and *qIGWS4-3*) were detected with minimal QEI effects and stable in different years. Among 111 QTLs,71 (63.40 %) QTLs were detected in both single and multiple environments.

**Conclusions:**

Three main QTL cluster regions associated with the 10 agronomic traits on chromosome 2H, 4H and 7H were detected. The QTLs for SMS, SLP, GP and GWP were located in the region near *Vrs1* on chromosome 2H. The QTLs underlying SMS, SPD and SLP were clustered on chromosome 4H. On the terminal of chromosome 7H, there was a QTL cluster associated with TGW, SPD, GWP and GWS. The information will be useful for marker-assisted selection (MAS) in barley breeding.

**Electronic supplementary material:**

The online version of this article (doi:10.1186/s12863-016-0409-y) contains supplementary material, which is available to authorized users.

## Background

Barley (*Hordeum vulgare* L.) is one of the world’s earliest domesticated crops and ranks the fourth cereal crop after maize, rice and wheat. It is widely used in many ways, including animal feed, malting and brewing. High-grain yield is the main breeding goal in developing cultivars. Due to most agronomic traits related to grain yield are controlled by quantitative trait loci (QTL), it is difficult to dissect the genetic and molecular basis of complex grain yield traits. Genome-analysis tools are useful for dissecting complex traits and manipulating determinants of multiple traits in breeding procedures [1–3].

QTL analysis has been widely applied to all crops including barley [4–7]. Since the first barley genetic map was constructed from RFLP marker, barley breeders have constructed many genetic maps using various genetic markers, including RFLP, AFLP, SSR and DArT [8–15]. These maps have been employed to identify, locate and estimate the phenotypic effects of QTLs underlying economically important traits [11, 12, 16–23]. Multiple environment trials, especially for grain yield traits, are commonly used to assess the performance of genotypes across a range of locations and years. Some QTLs are sensitive to environment and may have different effects in different years, with strong QTL × environment interaction (QEI). Therefore,identification QTL for grain yield trait in multiple environment and QTL × environment interaction to find crucial stable QTL is of vital importance for applying them in marker-assisted selection (MAS). With availability of genome sequence data and development of next generation sequencing (NGS) for rapid identification and scoring of genetic markers, single nucleotide polymorphism (SNP) markers have been widely used for constructing haplotype maps and genome-wide association studies [24–28]. Restriction site associated DNA (RAD) was first described as a tool for genetic mapping in fish and fungi by Baird et al. [29]. This newly developed sequencing technology can increase the data generated via short-read sequencing using restricted enzyme digested DNA to reduce complexity of genome compared to genome de novo sequencing and re-sequencing [29–32]. It has rapidly become a popular method for quick SNP discovery, linkage map construction [33, 34], and QTL mapping [35, 36].

Grain yield is directly determined by number of tillers per plant, number of grains per spike and thousand grain weight. Agronomic traits, such as spike density, main spike length, spike number per plant, grain weight per spike could indirectly affect the yield [5, 37–40]. QTLs underlying these important yield component traits are rarely analyzed in a cross between two-rowed and six-rowed barley. Huaai 11 is a new source of dwarfing discovered in our research group, and dwarf gene was mapped on the long arm of chromosome 7H [41]. With application of this population, our group constructed genetic map with 153 SSR markers to dissect phenotypic effect of QTL for agronomic traits, morphological and physiological traits of flag leaf [42, 43]. However, previous linkage map constructed based on 153 SSR markers is not able to provide precise and complete information about the numbers and location of QTL. The high density map is essential for precisely QTL identification and fine mapping of agronomic traits associated with grain yield [44]. The objective of this study was to use an ultra-high density SNP map containing 1894 SNP and 68 SSR markers to identify QTL underlying 10 agronomic traits related to grain yield.

## Methods

### Plant material and experimental design

The Huaai 11 was discovered from the barley landrace Dofu Bai Qing Ke and collected by Professor Sun Dongfa in 1993. Huadamai 6 is an elite brewing barley cultivar developed by Huazhong Agricultural University and available in China. The mapping population consists of 122 doubled haploid (DH) lines derived from a cross between the six-rowed dwarfing barley cultivar Huaai 11 (height is about 40 cm) and the two-rowed barley cultivar Huadamai 6 (height is about 85 cm) using anther culture [41]. The field trials were performed on the experimental farm of Huazhong Agricultural University, Wuhan (30°33’N) in five consecutive years (2009 to 2013). Each of the DH and parental lines were grown in three rows with a plot of lines of 1.5 m in length. The six seeds from each line were grown in each row, and space of the plant was 0.1 m.

### DNA isolation and genotype

Genomic DNA was extracted from seeding leaf using CTAB method, and treated with RNase to remove residual RNA. All lines from mapping population and two parents were genotyped at the Personal Biotechnology Co., Ltd (Shanghai, China). Genomic DNA was digested with the restriction endonuclease *Xma*I, a high fidelity restriction enzyme (New England Biolabs, USA), which recognizes an 6-nucleotide (nt) sequence (5'CCCGGG3'). The RAD library construction and DNA sequencing were done according to the protocol described by Baird et al. [29]. The DNA samples were performed in a single lane (library) of an Illumian Hiseq2000.

### Map construction

Using the procedure of Ramsey et al [45], Ren et al. [41, 42] genotyped 153 SSR markers. The RAD-seq markers were sorted using the MSTMAP software [46]. MSTMAP v4.3 software was used to generate individual group tree for the 122 lines. SNP markers with >15 % missing value were removed. JoinMap 4.0 was used to calculate the 1894 SNP and 68 polymorphic SSR markers order and genetic distance using Kosambi’s mapping function [47, 48]. Markers were assigned to seven linkage groups using a test for independence LOD score of 7.0, and ordered using the regression mapping algorithm. Two morphological markers, row number (*Vrs1*) and naked caryopsis (*Nud1*), were also integrated into the map.

### Phenotyping

After fully maturity, we randomly harvested four individual plants from each plot. Ten agronomic traits, spike number per plant (SP), main spike length (MSL), spikelet number per spike (SMS), spikelet number per plant (SLP), grain number per plant (GP), grain number per spikes (GS), grain weight per plant (GWP), grain weight per spikes (GWS), thousand grain weight (TGW) and spike density (SPD) were measured. Phenotypic data for those traits were measured as described by Ren et al. [42] except spike density (SPD) or spike (rachis) internodes length that was calculated using the number of fertile rachis nodes in a spike divided by length of main spike (cm). The mean values of twelve plants (four plants from each replicate x three replicates) sampled were subjected to statistical analysis. The phenotypic data of nine traits (except SPD) in 2009 and 2010 were from Ren et al. [42].

### Phenotypic data analysis

Correlation and QTL analyses were performed for the data from each year. Homogeneity of variance and normality of distribution were tested before analysis of variance (ANOVA) using the general linear model (GLM). All analyses were performed using IBM SPSS Statistics 19 software (http://www.spss.com). P value less than 0.05 was considered as significance.

### QTL analysis

The location of QTL and its genetic effect were detected through composite interval mapping (CIM) using QTL Cartographer version 2.5 [49]. After performing 1000 permutation test, a LOD (Likelihood of odd) threshold of 2.5 was used to declare putative QTLs [50]. Percentage of phenotypic variation explained and additive effect of each QTL were also calculated using QTL Cartographer 2.5. The confidence of interval was calculated using the two-LOD support interval, which was determined by finding the region on both sides of a QTL peak that corresponds to a decrease of 2 LOD score [51, 52]. The software MapChart 2.2 was used to draw QTL location on the map [53]. The putative QTLs were defined as ‘q’ + abbreviation trait name + detected QTL order on chromosome for CIM mapping. QTL in multiple environment interaction (QEI) mapping was conducted using the software IciMapping 4.0 (www.isbreeding.net). A LOD threshold ≥5.0 was set for declaring the QTL × environment interaction (QEI). The putative QTLs were defined as ‘q’ + ‘I’ + abbreviation trait name + detected QTL order on chromosome in multi-environment analysis using inclusive composite interval mapping (ICIM). QTLs linked to a target trait, which were stably identified from different years with clearly similar positions (overlapping intervals), were assumed to be the same one.

## Results

### Characteristics of agronomic traits

Table 1 showed 10 agronomic traits of the 122 individuals from the DH population and their parents in five consecutive years (year 2009–2013). The values of SP, MSL, GWP, GWS, and TGW in Huadamai 6 were higher than those in Huaai 11. The values of SMS, SLP, GS, GP and SPD were higher in Huaai 11 than those in Huadamai 6. The t-test showed that two parents were significant difference on all traits (*p* < 0.05) but GP. GP is determined by SP and GS. Because Huaai 11 had more grain number per spike and few spike number per plant, it could explain why GP in DH population was significant different although parents showed no significant different. Probabilistic of the distribution test showed nine traits (except SMS) displayed normal distribution with skewness and kurtosis of among -1 from 1 (Table 1), and SMS displayed bimodal distribution. Analysis of variance of the 122 DH lines and their parents showed highly significant genotype effect for the traits studied, and also a significant year effect for all traits except SPD and SMS (Additional file 1: Table S1). Genotype × environment interaction (GEI) was also significant for all traits. The variable coefficients ranged from 21.69–39.34 % in 2009, 20.46–34.78 % in 2010, 22.44–44.40 % in 2011, 21.40–40.57 % in 2012, and 16.06–39.49 % in 2013. Correlation coefficients among the 10 agronomic traits were given in (Additional file 2: Table S2). MSL, SMS, GP, GWP, SLP and GWS showed positive correlation with each other. SPD showed negative correlation with other traits. TGW showed significant negative correlation with SMS, SLP, GP and GS. SP showed significant positive correlation with GP and GWP, while GWS showed negative correlation with SP.

**Table 1:** The Statistics of 122 DH lines and their parents for ten agronomic traits in five years

Traits	Year	Huadamai6	Huaai11	ST	DH lines					
		Mean	mean		max	M in	Mean	Skewness	Kurtosis	CV(%)
SP	2009	12.75 ± 1.07	8.59 ± 0.67	0.003^b^	15.25	4.42	8.34 ± 0.16	0.32	0.92	21.69
	2010	10.67 ± 0.65	8.67 ± 0.31	0.014^a^	13.75	4.92	8.85 ± 0.16	0.78	0.04	20.46
	2011	6.50 ± 0.28	4.60 ± 0.88	0.007^b^	11.00	4.00	6.74 ± 0.15	0.50	−0.35	24.15
	2012	14.50 ± 1.38	9.27 ± 0.71	0.005^b^	21.06	6.83	12.82 ± 0.25	0.22	0.06	21.4
	2013	10.45 ± 0.68	9.82 ± 0.82	0.049^a^	13.72	5.50	9.83 ± 0.14	0.10	−0.40	16.06
MSL	2009	8.75 ± 0.33	4.34 ± 0.13	0.000^b^	9.91	3.44	5.93 ± 0.15	0.55	−0.58	27.46
	2010	11.25 ± 0.22	5.52 ± 0.10	0.000^b^	11.39	4.33	7.27 ± 0.16	0.31	−0.97	24.88
	2011	9.75 ± 0.46	5.12 ± 0.16	0.000^b^	11.20	3.78	6.53 ± 0.16	0.50	−0.69	27
	2012	10.39 ± 0.15	4.82 ± 0.14	0.000^b^	11.08	3.77	6.75 ± 0.17	0.46	−0.79	27.06
	2013	10.83 ± 0.21	4.88 ± 0.18	0.000^b^	12.25	4.17	6.87 ± 0.16	0.56	−0.54	26.01
SMS	2009	31.33 ± 1.14	50.50 ± 1.56	0.000^b^	80.50	18.00	50.67 ± 1.61	−0.54	−1.17	35.17
	2010	36.83 ± 0.83	63.00 ± 1.17	0.000^b^	91.00	21.00	59.88 ± 1.89	−0.70	−1.10	34.78
	2011	33.5 ± 0.50	57.00 ± 1.73	0.000^b^	93.00	16.00	53.42 ± 1.78	−0.35	−1.04	36.77
	2012	34.00 ± 0.84	56.40 ± 1.33	0.000^b^	95.00	19.01	55.41 ± 1.82	−0.51	−1.09	36.29
	2013	36.33 ± 0.41	58.00 ± 2.26	0.000^b^	91.00	23.00	57.68 ± 1.77	−0.62	−1.04	33.84
SLP	2009	365.50 ± 39.88	528.00 ± 38.05	0.026^a^	623.50	136.83	369.95 ± 12.28	0.02	−0.83	36.67
	2010	376.50 ± 18.84	520.00 ± 15.73	0.000^b^	765.50	150.50	481.36 ± 12.98	−0.32	−0.82	29.78
	2011	618.00 ± 39.87	219.00 ± 34.85	0.000^b^	643.50	83.50	325.93 ± 13.10	0.36	−0.71	44.4
	2012	551.75 ± 52.90	477.00 ± 25.94	0.024^a^	1244.00	180.39	670.79 ± 23.96	0.06	−0.97	39.46
	2013	374.17 ± 22.14	484.00 ± 29.78	0.007^b^	1009.50	184.83	545.22 ± 19.5	0.04	−0.91	39.49
GP	2009	288.50 ± 32.63	323.17 ± 28.46	0.024^a^	362.58	44.58	184.85 ± 6.32	0.44	−0.25	37.75
	2010	329.83 ± 20.48	375.50 ± 38.03	0.305	558.17	121.75	365.64 ± 9.48	−0.59	0.02	28.62
	2011	169.37 ± 11.63	160.25 ± 19.57	0.005^b^	523.00	78.00	257.32 ± 10.02	0.43	−0.59	43.01
	2012	415.40 ± 45.97	314.36 ± 31.78	0.065	956.50	128.42	431.47 ± 15.91	0.58	0.06	40.57
	2013	304.25 ± 12.44	371.25 ± 42.23	0.179	695.00	158.22	404.37 ± 11.61	−0.08	−0.74	31.71
GS	2009	22.17 ± 0.92	34.69 ± 3.37	0.003^b^	40.20	8.51	22.11 ± 0.68	0.50	−0.48	33.81
	2010	30.98 ± 0.50	43.03 ± 3.98	0.012^a^	66.13	16.34	43.69 ± 1.32	−0.56	−0.92	33.49
	2011	27.34 ± 0.83	38.16 ± 4.69	0.045^a^	77.00	13.15	38.56 ± 1.30	−0.02	−0.80	37.2
	2012	27.13 ± 1.31	32.21 ± 0.67	0.004^b^	71.74	12.09	33.83 ± 1.09	0.11	−0.53	35.47
	2013	28.32 ± 2.99	37.85 ± 2.13	0.004^b^	70.18	15.81	42.47 ± 1.21	−0.48	−0.99	31.36
GWP	2009	10.75 ± 1.34	4.29 ± 0.07	0.000^b^	10.17	1.00	4.94 ± 0.18	0.21	−0.30	39.34
	2010	17.23 ± 1.19	12.17 ± 0.65	0.000^b^	20.84	5.86	12.85 ± 0.29	−0.04	−0.66	25.08
	2011	6.49 ± 0.95	4.19 ± 0.54	0.001^b^	17.20	2.69	7.62 ± 0.27	0.94	0.76	39.85
	2012	12.99 ± 016	6.17 ± 0.63	0.002^b^	23.71	3.87	11.48 ± 0.38	0.61	0.17	36.02
	2013	17.03 ± 0.85	11.86 ± 1.36	0.000^b^	25.13	6.23	12.82 ± 0.33	0.52	0.41	28.21
GWS	2009	0.82 ± 0.05	0.47 ± 0.05	0.000^b^	0.97	0.19	0.58 ± 0.02	0.10	−0.62	31.03
	2010	1.61 ± 0.04	1.41 ± 0.07	0.018^a^	2.91	0.62	1.50 ± 0.04	0.30	−0.12	29.33
	2011	1.20 ± 0.09	0.99 ± 0.14	0.036^a^	2.06	0.41	1.12 ± 0.04	0.55	0.55	31.86
	2012	0.83 ± 0.06	0.67 ± 0.05	0.048^a^	1.47	0.35	0.89 ± 0.02	0.17	−0.86	29.21
	2013	1.58 ± 0.16	1.23 ± 0.04	0.016^a^	2.15	0.72	1.34 ± 0.03	0.22	−0.60	26.12
TGW	2009	36.69 ± 1.19	23.46 ± 0.81	0.003^b^	48.62	15.48	27.65 ± 0.60	0.93	0.55	24.16
	2010	52.05 ± 0.93	29.44 ± 0.72	0.000^b^	57.94	21.28	36.44 ± 0.82	0.55	−0.50	24.78
	2011	37.75 ± 4.40	26.14 ± 0.49	0.036^a^	57.06	15.87	31.63 ± 0.87	0.80	−0.21	30.4
	2012	30.18 ± 1.19	20.59 ± 1.40	0.000^b^	56.30	16.32	28.63 ± 0.78	0.98	0.41	30.19
	2013	55.94 ± 1.11	32.47 ± 0.68	0.000^b^	59.92	16.87	33.72 ± 0.95	0.66	−0.49	30.95
SPD	2009	3.59 ± 0.09	3.85 ± 0.08	0.049^a^	5.98	2.55	3.83 ± 0.08	0.53	−0.53	22.23
	2010	3.28 ± 0.71	3.81 ± 0.05	0.005^b^	6.29	2.50	3.68 ± 0.08	0.71	0.11	23.17
	2011	3.30 ± 0.09	3.70 ± 0.02	0.000^a^	5.88	2.36	3.64 ± 0.07	0.63	−0.10	22.44
	2012	3.24 ± 0.06	3.85 ± 0.10	0.000^b^	6.10	1.80	3.67 ± 0.08	0.62	−0.02	24.03
	2013	3.36 ± 0.06	3.98 ± 0.11	0.002^b^	7.35	2.32	3.74 ± 0.08	0.98	0.77	24.43

^a^Significant at the 5 % level, ^b^Significant at the 1 % level, respectively

*ST* Significance level, *CV* Coefficient of variation

### Marker genotyping

**Fig. 1:**
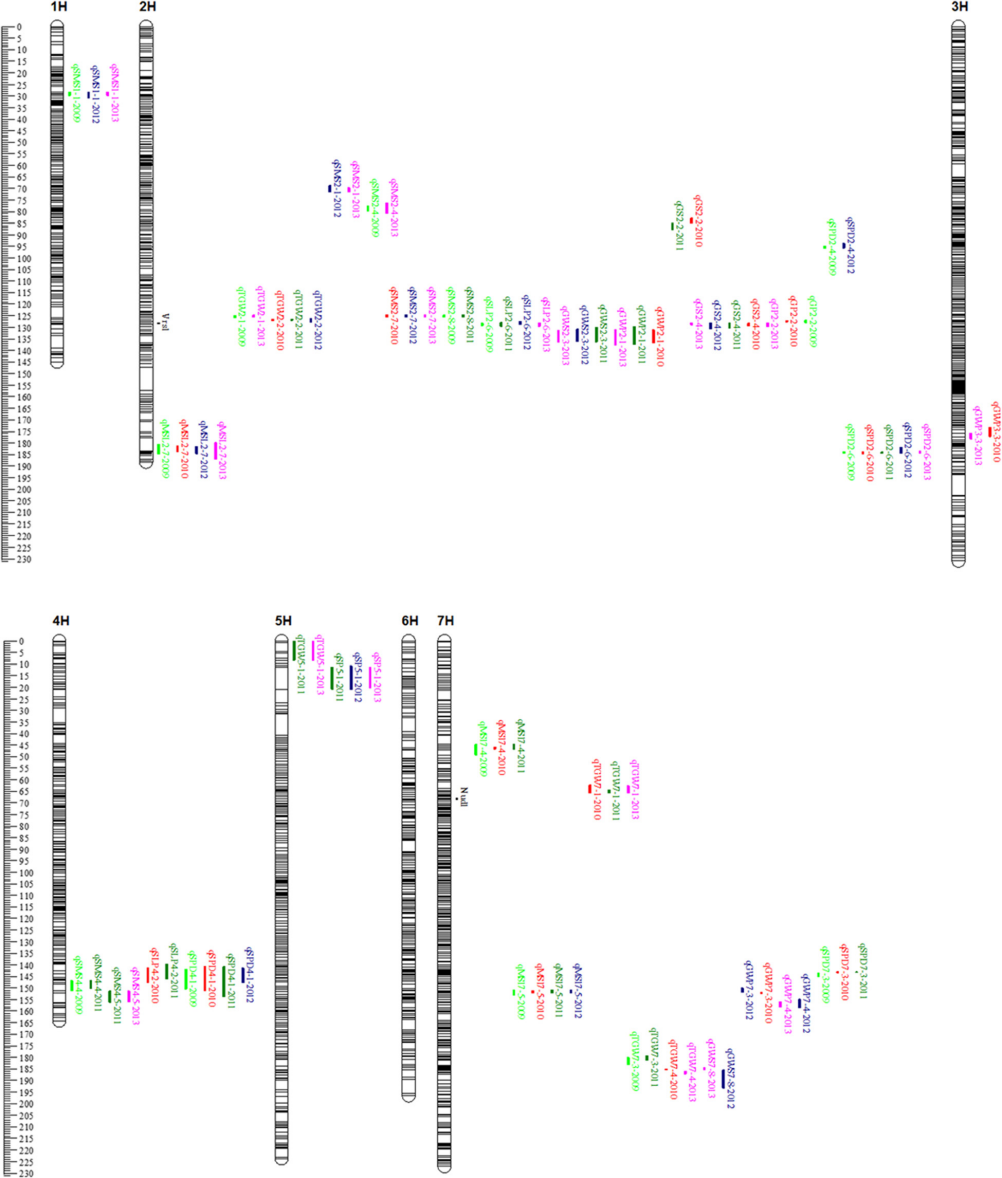
Chromosomes location of reliable QTL associated with 10 traits and two morphological traits (*black*). Genetic distance scale in centiMorgan (*cM*) is placed at left margin. Location of QTL is indicated for year 2009 (*Cadmium Green Pale*), 2010 (*orange red*), 2011 (*green*), 2012 (*purple*) and 2013 (*pink*)

A total of 4992 SNP polymorphic markers and 153 polymorphic SSR markers were used for constructing a high density SNP map. Two morphological markers, row number (*Vrs1*) and naked caryopsis (*Nud1*), were also integrated into the map. Linkage analysis positioned morphological marker *Vrs1* to the 0.72 cM marker interval flanked by SNP marker 2_522610509 and 2HL_34260490 (Fig. 1). The *Nud1* was positioned to the 0.50 cM marker interval flanked by SNP marker M_96819_188 and 7HL_29967547 (Fig. 1). The final map was composed of 1894 SNP and 68 SSR markers (the co-segregation of markers and non-linked markers were excluded). The total length of genetic map was 1375.8 cM with an averaged inter-marker distance of 0.70 cM.

### Genome mapping of SNP markers

To compare SNP marker distribution in coding and noncoding regions of the barley genome, we searched the locations of all 4992 SNP markers on reference genome. The high-quality SNP sequences were blasted against the comprehensive barley gene index in the NCBI (http://blast.ncbi.nlm.nih.gov/Blast.cgi). Using this database, 321 of 4992 (6.43 %) polymorphic SNP loci were aligned to multiple positions in gene index (Additional file 3: Table S3). High percentage of SNP sequences was positioned on candidate genes, suggesting that SNP markers were significantly rich in gene region.

### QTL analysis

A total of 221 QTLs for 10 observed agronomic traits were detected in five consecutive years using CIM mapping (Additional file 4: Table S4). The numbers of QTLs detected were 50, 48, 41, 41 and 41 for the year 2009 to 2013, respectively (Table 2).

**Table 2:** QTLs for the 10 observed agronomic traits on seven chromosomes

Trait	2009	2010	2011	2012	2013	Total
MSL	6	6	4	7	3	26
SMS	6	6	5	4	6	27
SLP	6	5	4	3	3	21
GP	3	3	2	4	4	16
GS	4	4	4	4	4	20
GWP	5	5	2	2	4	18
TGW	6	5	6	3	6	26
GWS	5	6	4	5	5	25
SP	3	4	4	4	3	18
SPD	6	4	6	5	3	24
Total	50	48	41	41	41	221

### Main spike length and spikelet number on main spike

For MSL, a total of 26 QTLs were identified in single environment in five consecutive years, its LOD value ranged from 2.80 to 39.79, and individually accounted for 1.16–52.72 % of the phenotypic variation (Additional file 4: Table S4). Three reliable QTLs were detected in more than one year (Table 3 and Fig. 1). Of them, the major QTL *qMSL2-7* was detected on chromosome 2H in four years, and accounted for 7.27–18.70 % of phenotypic variation with LOD value 7.24–17.32. *qMSL7-4* and *qMSL7-5* had positive additive effect on MSL and total explained 28.54–54.96 % of the phenotypic variation in three years (2009–2011) and four years (2009–2012), respectively. *qMSL7-4* and *qMSL7-5* were close to the SNP marker 7_542100185 and 7_249275418, respectively. The alleles from Huadamai6 increased MSL.

**Table 3:** Credible QTLs, their locations and effects for ten agronomic traits

Trait	QTL^a^	Chr.	LOD	R^2^(%)	Peak position	Nearest Marker	Interval^b^	Additive	Year
MSL	*qMsl2-7*	2	7.24–17.32	7.27–18.70	184.71	2_598551866	179.8–187.0	–	2009,2010,2011,2013
	*qMsl7-4*	7	4.07–4.71	1.93–3.07	46.01	7_542100185	44.7–49.2	+	2009,2010,2011
	*qMsl7-5*	7	23.23–39.79	25.47–52.72	151.51	7_249275418	150.8–153.0	+	2009,2010,2011,2012
SMS	*qSms1-1*	1	3.83–7.11	1.27–2.37	29.01	M_208488_950	28.2–30.9	+	2009,2012,2013
	*qSms2-1*	2	8.52–10.52	3.01–3.34	69.01	2_426554428	68.8–71.5	+	2012,2013
	*qSms2-4*	2	8. 21–10.28	2.96–3.41	79.01	M_223185_603	76.3–80.8	+	2009,2013
	*qSms2-7*	2	54.54–67.58	72.76–82.22	125.21	2HL_22930294	124.6–125.6	+	2009,2010,2011,2012,2013
	*qSms4-4*	4	7.03–7.39	2.35–3.35	148.41	4HS_28564347	146.7–151.3	+	2009,2011
	*qSms4-5*	4	4.97–5.02	1.65–2.83	154.01	4HS_9277108	151.3–156.2	+	2012,2013
SPD	*qSpd2-4*	2	20.37–20.63	4.38–3.99	94.41	M_230635_804	93.7–95.8	+	2009,2012
	*qSpd2-6*	2	39.52–39.98	16.85–23.94	184.11	2_598509820	181.8–184.6	+	2009,2010,2011,2012,2013
	*qSpd4-1*	4	30.52–30.83	1.72–2.92	145.51	4HS_32009949	140.8–153.8	+	2009,2010,2011,2012
	*qSpd7-3*	7	31.09–31.17	46.97–57.06	143.81	7_379313804	143.4–145.1	–	2009,2010,2011
SP	*qSp5-1*	5	2.55–6.00	6.18–20.32	13.31	5_6313908	10.8–20.9	+	2011,2012,2013
SLP	*qSlp2-6*	2	5.62–32.59	16.70–64.20	128.71	2HL_34260490	127.8–129.6	–	2009,2011,2012,2013
	*qslp4-2*	4	3.40–5.15	4.72–5.05	143.71	4_28741355	139.6–146.0	+	2010,2011
TGW	*qTgw2-1*	2	28.10–48.43	44.03–61.78	125.21	2HL_22930294	124.7–126.0	+	2009,2013
	*qTgw2-2*	2	24.08–49.64	43.45–58.63	127.01	2HL_22930005	126.2–127.7	+	2010,2011,2012
	*qTgw5-1*	5	2.69–3.32	1.39–1.56	2.91	5_54803	0–8.4	+	2011,2013
	*qTgw7-1*	7	6.54–18.45	3.02–16.22	64.81	7HL_28107982	62.3–65.8	+	2010,2011,2013
	*qTgw7-3*	7	5.47–10.51	3.00–10.99	181.11	7_100916612	180.0–183.3	+	2009,2011
	*qTgw7-4*	7	10.50–13.16	5.42–6.64	186.21	7_74542989	185.9–187.2	+	2010,2013
GP	*qGp2-2*	2	5.65–33.76	11.70–57.24	128.71	2HL_34260490	128.0–129.6	+	2009,2010,2013
GS	*qGs2-2*	2	4.83–5.64	1.83–4.73	85.91	M_207663_1931	85.0–87.7	+	2010,2011
	*qGs2-4*	2	21.68–58.64	41.14–79.50	128.71	2HL_34260490	128–130.6	+	2010,2011,2012,2013
GWP	*qGwp2-1*	2	3.75–5.59	6.68–11.72	133.01	2_534686550	129.6–137.4	+	2010,2011,2013
	*qGwp3-3*	3	2.73–5.64	6.01–6.85	174.31	3HL_45008254	173.1–177.3	±	2010,2013
	*qGwp7-3*	7	7.72–21.59	11.85–40.82	151.51	7_249275418	150.0–151.9	+	2010,2012
	*qGwp7-4*	7	4.35–13.38	7.11–28.85	157.11	M_231959_191	154.9–158.7	+	2012,2013
**GWS**	*qGws2-3*	2	6.48–9.34	12.44–16.83	133.01	2_534686550	130.0–136.3	–	2011,2012,2013
	*qGws7-8*	7	2.92–4.06	4.08–6.50	186.71	7_92359522	185.4–193.3	+	2012,2013

^a^Credible QTLs which was detected more than one year

^b^2-LOD confidence interval which was determined by finding the region on both sides of a QTL peak that corresponds to a decrease of 2 LOD score

For SMS, a total of 27 QTLs were detected and dispersed on chromosome 1H, 2H, 4H and 7H. Each LOD value ranged from 2.58 to 87.42, and individually accounted for 1.07–82.22 % phenotypic variation (Additional file 4: Table S4). Six reliable QTLs were detected in more than one year (Table 3). *qSMS2-1* (year 2012 and 2013), *qSMS2-4* (year 2009 and 2013), *qSMS4-4* (year 2009 and 2011) and *qSMS4-5* (year 2012 and 2013) were detected in different two years and showed minor effects. *qSMS2-7* had highest effect on SMS, and accounted for 72.76–82.22 % of phenotypic variation. *qSMS2-7* was close to the SNP marker 2HL_22930294, and the favorable alleles came from Huaai11 increasing SMS. On chromosome 1H, *qSMS1-1* had positive additive effect on SMS and explained 1.27–2.37 % of phenotypic variation in three years (year 2009, 2012 and 2013).

### Spike density

A total of 24 QTLs were detected underlying SPD, whose LOD value ranged from 2.94 to 44.73, and individually accounted for 1.34–85.06 % of phenotypic variation (Additional file 4: Table S4). Four reliable QTLs were detected on chromosome 2H, 4H and 7H (Table 3). Two QTLs (*qSPD2-4* and *qSPD2-6*) on chromosome 2H had positive additive effect on SPD. *qSPD2-6* was detected in five years, and explained 16.85–23.94 % of phenotypic variation, while the *qSPD2-4* was detected in year 2009 and 2012, explaining 4.38 % and 3.99 % of phenotypic variation. The *qSPD4-1* on chromosome 4H was detected in four years (year 2009 to 2012), and had the effect on increasing SPD and explained 1.72–2.92 % of phenotypic variation. The *qSPD7-3* on chromosome 7H had main effect on SPD, which was detected in three years (2009 to 2011), and accounted for 46.97–57.06 % of phenotypic variation.

### Spike numbers per plant

For SP, a total of 18 QTLs were detected in five consecutive years, whose LOD value ranged from 2.55 to 19.22 and individually accounted for 3.50–51.97 % of phenotypic variation (Additional file 4: Table S4). Only one reliable QTL *qSP5-1* on chromosome 5H was identified for SP in year 2011, 2012 and 2013, with effect on increasing spike number per plant and explained 6.18–20.32 % of phenotypic variation (Table 3).

### Spikelet numbers per plant

A total of 18 QTLs influencing SLP were identified, individually explaining 3.50–51.97 % of phenotypic variation with LOD value 2.55–19.22 (Additional file 4: Table S4). Two reliable QTLs were detected in more than one year (Table 3). *qSLP2-6* has highest effects on SLP, and was detected in four years (2009, 2011, 2012 and 2013), and accounted for 16.70–64.20 % of phenotypic variation. *qSLP2-6* was close to the marker 2HL_34260490 with favorable alleles from Huaai11 for increasing SLP. *qSLP4-2* on chromosome 4H had positive additive effect on SLP.

### Thousand grain weight

For TGW, a total of 26 QTLs were detected in five consecutive years, whose LOD ranged from 2.69 to 49.64 and individually accounted for 1.39–61.78 % of phenotypic variation (Additional file 4: Table S4). Six reliable QTLs underlying TGW were detected in more than one year and were mapped on chromosome 2H, 5H and 7H (Table 3). Two QTLs (*qTGW*2-1 and *qTGW2-2*) were close to the *Vrs 1* marker, and their additive effects were constancy in five years. *qTGW2-1* was detected in year 2009 and 2013, and explained 44.03 % and 61.78 % of phenotypic variation, respectively, while *qTgw2-2* was detected in year 2010, 2011 and 2012, explaining 43.45 % to 58.63 % of phenotypic variation. *qTGW5-1* on chromosome 5H, was identified in year 2011 and 2013, with effect on decreasing thousand grain weight and explained 1.56 % and 1.39 % of phenotypic variation, respectively. Three QTLS (*qTGW7-1*, *qTGW7-3* and *qTGW7-4*) on chromosome 7H had positive effect on TGW. *qTGW*7-1 was detected in three years, explaining 3.02–16.22 % of phenotypic variation. *qTGW7-3* was detected in year 2009 and 2011, and explained 10.99 % and 3.00 % of phenotypic variation, respectively. *qTGW7-4* was detected in year 2010 and 2013, and accounted for 6.64 % and 5.42 % of phenotypic variation, respectively.

### Grain number per plant and grain number per spike

A total of 16 QTLs underlying GP were detected on chromosome 2H, 4H, 5H, 6H and 7H, individually explaining 2.54–57.24 % of phenotypic variation with LOD value 2.69–33.76 (Additional file 4: Table S4). One reliable QTL (*qGP2-2*) on chromosome 2H was identified for GP in three years, with effect on increasing grain number per plant. *qGP2-2* explained 11.70–57.24 % of phenotypic variation (Table 3).

For GS, a total of 20 QTLs were detected in five consecutive years, whose LOD value ranged from 2.63 to 58.64 and individually accounted for 1.74–79.50 % of phenotypic variation (Additional file 4: Table S4). Two reliable QTLs were detected on chromosome 2H in more than one year (Table 3). *qGS2-2* was detected in two years and accounted for 1.83 % and 4.73 % of phenotypic variation in 2010 and 2011, respectively, with favorable alleles from Huadamai 6. *qGS2-4* was detected in four years and accounted for 41.14–79.50 % of phenotypic variation in 2010 to 2013, respectively, with favorable alleles from Huadamai6. *qGS2-4*, close to the marker 2HL_34260490, had main effect on decreasing GS.

### Grain weight per plant and grain weight per spike

For GWP, a total of 18 QTLs were detected in five consecutive years, whose LOD value ranged from 2.52 to 21.59 and individually accounted for 3.09–40.82 % of phenotypic variation (Additional file 4: Table S4). Four reliable QTLs underlying GWP were detected in more than one year (Table 3). *qGWP3-3* on chromosome 3H was identified in two years, explained 6.85 % and 6.01 % of phenotypic variation in 2010 and 2013, respectively. *qGWP2-1* on chromosome 2H was identified in three years, with effect on increasing grain weights per plant, and explained 6.68 % to 11.72 % of phenotypic variation. Two QTLs (*qGWP7-3* and *qGWP7-4*) on chromosome 7H had positive additive effect on grain weight per plant. *qGWP7-3* accounted for 40.82 % and 11.85 % of phenotypic variation in 2010, and 2012, while *qGWP7-4* accounted for 7.11 % and 28.85 % of phenotypic variation in 2012 and 2013, respectively.

For GWS, a total of 25 QTLs were detected in five consecutive years, whose LOD value ranged from 2.67 to 26.59 and individually accounted for 2.08–40.82 % of phenotypic variation (Additional file 4: Table S4). Two QTLs were detected in more than one year (Table 3). *qGWS2-3* on chromosome 2H had effect on decreasing GWS, which was detected in three years and explained 12.44–16.83 % of phenotypic variation. *qGWS7-8* on chromosome 7H was detected in two years, accounted for 4.08 % and 6.50 % of phenotypic variation in 2012 and 2013, respectively.

### QTL × environment interaction (QEI) analysis

A total 71 of 111 significant QTLs for 10 agronomic traits were detected using both single and multiple environment analyses over five environments (Additional file 5: Table S5). Among them, 31 QTLs had major effects on their target traits, in which 12 QTLs were also major QTLs identified in single environment.

For MSL, 18 QTLs were detected across the five environments: one each on chromosome 3H and 6H,two each on chromosome 1H and 4H, four and eight on chromosome 7H and 2H, respectively. Eleven QTLs were detected in both single and multiple environments mapping while seven QTLs were only detected in multiple environments mapping analysis (Additional file 5: Table S5). Four QTLs (*qIMSL3-1, qIMSL4-1, qIMSL4-2 and qIMSL6-1*) were relatively stable, whose LOD_A_ value ranged from 4.81 to 6.02, and LOD_AE_ ranged from 0.54 to 1.97. Three QTLs (*qIMSL2-8*, *qIMSL7-3 and qIMSL7-4*) were detected in more than one year. The highest main-effect QTL underlying MSL identified in five years was *qIMSL2-8*, its LOD_A_ and LOD_AE_ was 88.78 and 42.64, respectively. This major QTL *qIMSL2-8* was environment-specific, close to *qMSL2-7* detected using CIM mapping.

A total 16 QTLs influencing SMS were identified across the five environments: one each on chromosome 1H and 3H,two each on chromosome 4H, four on chromosome 7H and eight on chromosome 2H. Eight QTLs were detected in both single and multiple environments mapping while other eight QTLs were only detected in multiple environments mapping analysis (Additional file 5: Table S5). Four QTLs (*qISMS2-3*, *qISMS2-6*, *qISMS4-1* and *qISMS4-2*) were detected in more than one year. *qISMS7-1* was relatively stable, its LOD_A_ and LOD_AE_ was 7.61 and 1.35, respectively. Three QTLs (*qISMS2-1*, *qISMS2-2* and *qISMS4-2*) had strong QEI. LOD_A_ ranged from 1.07 to 1.73, and LOD_AE_ ranged from 5.02 to 5.17. The highest main-effect QTL underlying SMS identified in five years was *qISMS2-6*, its LOD_A_ and LOD_AE_ was 150.54 and 248.14, respectively. This major QTL *qISMS2-6* was environment-specific, close to *qSMS2-7* detected using CIM mapping.

For SPD, 15 QTLs were identified across the five environments: one each on chromosome 3H, 5H and 6H,three on chromosome 7H and nine on chromosome 2H. Ten QTLs were detected in both single and multiple environments mapping analyses, while five QTLs were only detected using multiple environments mapping (Additional file 5: Table S5). Three QTLs (*qISPD2-6*, *qISPD2-9* and *qISPD5-1*) were detected in more than one year. Two QTLs (*qISPD2-7* and *qISPD7-1*) were relatively stable, whose LOD_A_ ranged from 4.33 to 5.29, and LOD_AE_ ranged from 1.07 to 1.67. *qISPD2-5* had strong QEI, its LOD_A_ and LOD_AE_ was 1.76 and 9.68, respectively. The highest main-effect QTL underlying SPD identified was *qISPD2-9*, its LOD_A_ and LOD_AE_ was 66.82 and 56.50, respectively. This major QTL *qISPD2-9* was environment-specific, close to *qSPD2-6* detected using CIM mapping.

For SP, 5 QTLs were identified across the five environments: one on chromosome 5H, two each on chromosome 2H and 7H. All QTLs were detected using both single and multiple environments mapping analyses (Additional file 5: Table S5). Only *qISP5-1* was detected in more than one year. *qISP2-1* had strong QEI, its LOD_A_ and LOD_AE_ was 1.24 and 4.83, respectively. Other four QTLs were identified with environment-specific effect. The highest main-effect QTL underlying SP identified was *qISP7-2*, its LOD_A_ and LOD_AE_ was 6.39 and 20.46, respectively.

For SLP, 4 QTLs were identified across the five environments: one each on chromosome 4H and 7H, two on chromosome 2H. All QTLs were detected in both single and multiple environments mapping analyses (Additional file 5: Table S5). Only *qISLP2-2* was detected in more than one year. *qISLP2-1* had strong QEI, its LOD_A_ and LOD_AE_ was 0.33 and 5.52, respectively. Other three QTLs were identified with environment-specific effect. The highest main-effect QTL underlying SP identified in four years was *qISLP2-2*, its LOD_A_ and LOD_AE_ was 26.16 and 61.52, respectively. This major QTL *qISLP2-2* was environment-specific, close to *qSLP2-6* detected using CIM mapping.

For TGW, 14 QTLs were identified across the five environments: one on chromosome 3H,two each on chromosome 1H, 2H, 4H and 5H, five on chromosome 7H. Ten QTLs were detected using both single and multiple environments mapping analyses, while four QTLs were only detected using multiple environments mapping (Additional file 5: Table S5). Six QTLs (*qITGW2-1*, *qITGW2-2*, *qITGW5-1*, *qITGW7-1, qITGW7-4* and *qITGW7-5*) were detected in more than one year. *qITGW3-1* was relatively stable, its LOD_A_ and LOD_AE_ was 4.14 and 1.15, respectively. *qITGW4-1* had strong QEI, its LOD_A_ and LOD_AE_ was 0.50 and 6.96, respectively. The highest main-effect QTL underlying TGW identified was *qITGW2-2*, its LOD_A_ and LOD_AE_ was 91.64 and 89.10, respectively. This major QTL *qITGW2-2* was environment-specific, close to *qTGW2-2* detected using CIM mapping.

For GP, 5 QTLs were identified across the five environments: one on chromosome 2H,two each on chromosome 4H and 7H. Three QTLs were detected using both single and multiple environments mapping analyses, while two QTLs (*qIGP4-1* and *qIGP7-2*) were only detected using multiple environments mapping (Additional file 5: Table S5). *qIGP7-1* had strong QEI, its LOD_A_ and LOD_AE_ was 1.64 and 13.43, respectively. The highest main-effect QTL underlying GS identified in four years was *qIGP2-1*, its LOD_A_ and LOD_AE_ was 56.99 and 36.23, respectively. This major QTL *qIGP2-1* was environment-specific, close to *qGP2-1* detected using CIM mapping.

For GS, 11 QTLs were identified across the five environments: one on chromosome 3H, three each on chromosome 1H and 7H, four on chromosome 2H. Eight QTLs were detected using both single and multiple environments mapping analyses, while three QTLs were only detected using multiple environments mapping (Additional file 5: Table S5). Two QTLs (*qIGS2-2* and *qIGS2-3*) were detected in more than one year. Three QTLs (*qIGS7-1*, *qIGS7-2* and *qIGS7-3*) had strong QEI, whose LOD_A_ ranged from 0.91 to 1.71, and LOD_AE_ ranged from 5.69 to 9.35. The highest main-effect QTL underlying GS identified in four years was *qIGS2-3*, its LOD_A_ and LOD_AE_ was 86.98 and 110.08, respectively. This major QTL *qIGS2-3* was environment-specific, close to *qGS2-4* detected using CIM mapping.

For GWP, 8 QTLs were identified across the five environments: one on chromosome 6H, two each on chromosome 2H and 3H, three on chromosome 7H. Four QTLs were detected using both single and multiple environments mapping analyses, while four QTLs were only detected using multiple environments mapping (Additional file 5: Table S5). Only *qIGWP2-1* was detected in more than one year. *qIGWP7-2* had strong QEI, LOD_A_ and LOD_AE_ was 0.95 and 20.70, respectively. The highest main-effect QTL underlying GWP was *qIGWP6-1*, its LOD_A_ and LOD_AE_ was 12.25 and 28.30, respectively.

For GWS, 15 QTLs were identified across the five environments: one each on chromosome 3H, 5H and 6H, three each on chromosome 2H and 4H, seven on chromosome 7H. Eight QTLs were detected using both single and multiple environments mapping analyses, while seven QTLs were only detected using multiple environments mapping (Additional file 5: Table S5). Two QTLs (*qIGWS2-3* and *qIGWS7-6*) were detected in more than one year. *qIGWS4-3* was relatively stable, its LOD_A_ and LOD_AE_ was 4.59 and 1.20, respectively. The highest main-effect QTL underlying GWS was *qIGWS7-2*, its LOD_A_ and LOD_AE_ was 85.71 and 2.32, respectively.

## Discussion

### Advantages of mapping agronomic trait with SNP markers

Sequence-based genotyping method could provide high quality SNPs for constructing an ultra-high density genetic map. In the present study, a high density genetic linkage map containing 1894 SNP markers and 68 SSR markers covering 1375.8 cM were used to identify quantitative trait loci. In the previous study using 153 SSR markers to dissect agronomic traits, we detected 12 QTLs for eight agronomic traits, one for SP, two for MSL, one for SLP, two for GP,one for GS, two for GWP, two for GWS, and one for TGW in 2009 [42]. In present study, we detected 35 QTLs for those traits: three for SP, six for MSL, six for SLP, four for GS, five for GWP, five for GWS and six for TGW with SNP markers (Table 2). In 2010, we detected 6 QTLs for four agronomic traits: two for MSL, one for GWP, one for GWS, two for TGW [42]. In present study, we detected 38 QTLs for those traits: three for SP, six for MSL, six for SLP, three for GP, four for GS, five for GWP, five for GWS and six for TGW with SNP markers (Table 2). It demonstrated advantage of SNP in detecting power and resolution relative to SSR map. The SNP markers were distributed across all seven linkage groups, with polymorphic loci covering both coding and noncoding regions in the barley genome. The accuracy and quality of the SNP markers identified will provide more associated markers for marker-assistant breeding (MAS) [23, 28, 34, 54, 55].

### QTL affecting grain yield and its related agronomic traits

In barley, main spike length (MSL) and spike density (SPD) are two of the most important spike morphological traits. They not only affect grain yield potential, but also the yield of malt extract [56]. In our study, three credible QTLs were detected for MSL using CIM mapping (Table 3). *qMSL7-5* was detected in four years, indicating this QTL was not affected by environment. This QTL was also mapped on the same position with the dwarf gene *btwd1* that we had previously studied, which was linked to the SSR marker Bmac0031 and Bmac0167 [41]. This QTL is likely same to the QTL *Qel 7.1* on the chromosome 7H reported by Li et al. [57] , and different from the QTL reported by Sameri et al. [58]. On the terminal of chromosome 2HL, *qMSL2-7* was close to the 2_598509820 marker, and is likely the same locus on 2HL reported by Xue et al. [59]. The QTL, *qMSL1-2* (Additional file 4: Table S4), close to the marker 1H_10863328 and SSR markers Bmac90 and EBmac501 on chromosome 1H, is likely different from the *Qsl-tera_1H* reported by von Korff et al. [60]. The SSR marker Bmac90 was associated with days to heading on chromosome 1H [61]. QTLs for main MSL were reported to be located on all seven linkage groups [16, 22, 56–58, 62, 63].

QTLs conferring SPD were reported on 2H, 3H and 7H [57, 63–65]. On chromosome 2H, there were two significant QTLs (*qSPD2-4* and *qSPD2-6*). *qSPD2-6* was close to the SSR marker Ebmag793, which is near to the marker EBmac415 inferred from http://wheat.pw.usda.gov/GG3/, and is likely the same loci on chromosome 2HL as reported by Sameri et al. [57]. *qSPD2-4* is likely new one for spike density (SPD). *qSPD4-1* was detected in four years and close to the marker M_153521_1223, and is likely a new QTL. *qSPD7-3* had main effect on spike density, is close to the marker 7_379313804 and SSR marker Bmac0031 on chromosome 7HS, and is likely the same loci reported by Sameri et al. and Shahinnia et al. [57, 65].

The spikelet number on main spike (SMS) and spikelet number per plant (SLP) are closely correlated. As expected, two QTL regions (*qSMS2-7* and *qSLP2-6*, *qSMS4-4* and *qSLP4*), located nearby to each other on the chromosome were detected (Table 3). QTLs conferring SMS were previously reported on chromosome 1H, 2H, 5H and 7H [19, 66]. The main effect QTL *qSMS2-7* associated with the marker 2HL_22930294 was close to the morphological marker *Vrs1* on chromosome 2H, explaining 72.76 % to 82.22 % of phenotypic variance. The *Vrs1* locus, which primarily determines the row type of spike, has a pleiotropic effect on many agronomic characters such as the number of rachis nodes, spike length, stem length, thousand grain weight, fusarium head blight (FHB) resistance and heading date [18, 19, 60, 67–69]. *qSMS4-4* and *qSMS4-5* were close to the SSR marker HVM40, and are likely new loci underlying SMS. QTLs for SLP were detected on chromosome 2H and 4H, and QTLs for controlling spikelet number were rarely reported in barley.

For grain number per plant (GP) and grain numbers per spike (GS), there was a common QTL on chromosome 2H (Table 3). This QTL was close to the SNP marker 2HL_22930294, which was nearby the morphological marker *Vrs1*. QTLs for grain number per plant are reported to be located on chromosomes 1H [17, 22], 2H [56, 62, 63], 3H and 4H [70].

Only one credible QTL *qSP5-1* for spike number per plant was detected and close to the SNP marker 5_6313908 and SSR marker GBM1176. The SSR marker GBM1176 tightly linked to SNP marker scssr07106 that was associated with a main QTL for abiotic stress on chromosome 5H [11]. QTLs underlying spike numbers per plant were previously reported on the 1H, 2H, 5H, 6H and 7H [22, 37, 66].

Thousand grain weight (TGW) is one of the major yield components having direct effect on final yield. As shown in Table 3, the SNP associated with TGW were previously reported on seven linkage groups [5, 17, 37, 59, 63, 71, 72]. The main effect QTL *qTGW2-1* and *qTGW2-2* associated with the marker 2HL_22930294 and 2HL_22930005, respectively, were close to the morphological marker *Vrs1*, explaining 43.45 % to 61.78 % of phenotypic variance. *qTGW5-1* on the terminal of 5HS is likely new QTL and different from previously reported [17]. The *qTGW7-1* on chromosome 7H was close to the marker 7HL_28107982, which is in the same region close to the morphological marker *Nud1* on the chromosome 7H. This QTL seems to be a new one and different from the QTLs on chromosome 7H reported by Pillen et al. [17] and von Korff et al. [59].

QTLs conferring grain yield were reported to be located on all seven chromosomes [59, 62, 70, 73]. QTLs for GWP and GWS were mainly detected on chromosome 2H and 7H (Table 3). *qGWP2-1* and *qGWS2-3* were at the same region on chromosome. *qGWP7-3* and *qGWP7-4* are likely same ones reported by [17] on short arm of chromosome 7H.

### QTL × environment interaction (QEI)

Because of genetic variation in complex traits including environment × genotype interaction effect, some QTLs were often detected with small effects and low stability across different environments. Thus, consistency of QTLs across different years and environments is a major concern for marker-assisted selection to improve complex traits, especially for yield traits. In this study, we used multi-environment analysis to detect QTLs with QEI effects and find stable QTLs across different environments for 10 agronomic traits related to yield. Nine QTLs (*qIMSL3-1, qIMSL4-1, qIMSL4-2, qIMSL6-1, qISMS7-1, qISPD2-7, qISPD7-1, qITGW3-1* and *qIGWS4-3*) were detected with minimal QEI effects and stable across different years. These QTLs may be better ones for MAS-based breeding. Compared the QTLs detected using single analysis with CIM mapping and multi-environment analysis with ICIM mapping, we found that 71 QTLs identified using CIM mapping were also detected using ICIM mapping, especially for QTL detected in more than one environment. For instance, the main effect QTL (*qMSL2-7*) for MSL identified using CIM mapping located at 184.71 cM on the chromosome 2H, was also detected using ICIM mapping. However, some QTLs with special environment interaction detected using ICIM mapping were not detected using CIM.

### Clustering of the detected QTLs on linkage groups

A number of QTLs underlying the agronomic traits were detected in this cross. Some were highly genetically correlated and mapped to similar positions (Fig. 1). There are 11 genomic regions controlling these traits studied here: one on chromosome 1H, three on the chromosome 2H, one on chromosome 3H, one on chromosome 4H, one on chromosome 5H and four on chromosome 7H. A cluster of QTL for SMS, GS, SLP, GP, GWP and TGW was found on chromosome 2H (Fig. 1). This cluster was close to the morphological marker *Vrs1*. Some QTLs for SPD and MSL were located on the same region, such as on long arm of chromosome 2H and on chromosome 7H, but some mapped independently. Other traits like SMS, SPD and SLP were also clustered on chromosome 4H.

## Conclusions

A number of QTLs associated with multiple traits were found on certain chromosome regions, and some of these traits were significantly correlated with each other. The two parents used for mapping population construction are different row types (six-rowed dwarfing barley cultivar Huaai 11 and two-rowed barley cultivar Huadamai 6). A lot of QTLs detected were close to the morphological marker *Vrs1* region, for example *qSMS2-7*, *qSLP2-6*, *qTGW2-1*, *qTGW2-2*, *qGP2-2*, *qGS2-4* and *qGWP2-1*. On the terminal of chromosome 7H, the QTLs for MSL, SP and SPD were clustered together. The SSR maker GBM1149 and SNP marker 2_598509820 could be used for marker-assisted selection for these three traits.

## Abbreviations

DH, Double haploid; RAD, Restriction site associated DNA; GP, Grain number per plant; GWP, Grain weight per plant; GS, Grain number per spike; TGW, Thousand grain weight; GWS, Grain weight per spike; SPD, pike density; ICIM, Inclusive composite interval mapping; LOD, Likelihood of odd; CIM, Composite interval mapping; QEI, QTL × environment interaction; MSL, Length of main spike; QTL, Quantitative trait locus; MAS, Marker assisted selection; SMS, Spikelet number on main spike; SLP, Spikelet number per plant; SNP, single nucleotide polymorphism; SP, Spike numbers per plant.SM, Spike morphology

## Additional files

Additional file 1: Table S1. Analysis of variance on 10 agronomic traits in DH lines. (XLSX 8 kb) Additional file 2: Table S2: Correlation coefficients among the 10 observed agronomic traits. (XLSX 9 kb) Additional file 3: Table S3: Genome mapping of SNP sequence markers on *Hordeum* gene index in the NCBI. (XLSX 37 kb) Additional file 4: Table S4: List of 221 QTLs detected for 10 agronomic traits, their position, likelihood of odd (LOD), additive effect, explained variance and 2-LOD confidence interval. (XLSX 27 kb) Additional file 5: Table S5: Putative QTLs for 10 agronomic traits through multiple environments QTL mapping. (XLSX 25 kb)
